# Parallel randomized controlled feasibility trials of the “Active Brains” digital intervention to protect cognitive health in adults aged 60–85

**DOI:** 10.3389/fpubh.2022.962873

**Published:** 2022-09-20

**Authors:** Rosie Essery, Sebastien Pollet, Katherine Bradbury, Max J. Western, Elisabeth Grey, James Denison-Day, Kirsten A. Smith, Victoria Hayter, Joanne Kelly, Jane Somerville, Beth Stuart, Taeko Becque, Jin Zhang, Joanna Slodkowska-Barabasz, Fiona Mowbray, Anne Ferrey, Guiqing Yao, Shihua Zhu, Tony Kendrick, Simon Griffin, Nanette Mutrie, Sian Robinson, Helen Brooker, Gareth Griffiths, Louise Robinson, Martin Rossor, Clive Ballard, John Gallacher, Shanaya Rathod, Bernard Gudgin, Rosemary Phillips, Tom Stokes, John Niven, Paul Little, Lucy Yardley

**Affiliations:** ^1^University of Southampton, Southampton, United Kingdom; ^2^NIHR ARC Wessex, Southampton, United Kingdom; ^3^University of Bath, Bath, United Kingdom; ^4^University of Bristol, Bristol, United Kingdom; ^5^Queen Mary University of London, London, United Kingdom; ^6^Oxford Brookes University, Oxford, United Kingdom; ^7^Kings College London, London, United Kingdom; ^8^University of Oxford, Oxford, United Kingdom; ^9^University of Leicester, Leicester, United Kingdom; ^10^University of Cambridge, Cambridge, United Kingdom; ^11^University of Edinburgh, Edinburgh, United Kingdom; ^12^Newcastle University, Newcastle, United Kingdom; ^13^University of Exeter, Exeter, United Kingdom; ^14^NIHR Southampton Clinical Trials Unit, University of Southampton and University Hospital Southampton NHS Foundation Trust, Southampton, United Kingdom; ^15^University College London, London, United Kingdom; ^16^Department of Psychiatry, University of Oxford, Oxford, United Kingdom; ^17^Southern Health NHS Foundation Trust, Southampton, United Kingdom; ^18^Patient and Public Involvement Contributor, University of Southampton, Southampton, United Kingdom

**Keywords:** dementia prevention, behavior change, physical activity, cognitive training, healthy eating

## Abstract

**Introduction:**

Multidomain interventions to address modifiable risk factors for dementia are promising, but require more cost-effective, scalable delivery. This study investigated the feasibility of the “Active Brains” digital behavior change intervention and its trial procedures.

**Materials and methods:**

Active Brains aims to reduce cognitive decline by promoting physical activity, healthy eating, and online cognitive training. We conducted 12-month parallel-design randomized controlled feasibility trials of “Active Brains” amongst “lower cognitive scoring” (*n* = 180) and “higher cognitive scoring” (*n* = 180) adults aged 60–85.

**Results:**

We collected 67.2 and 76.1% of our 12-month primary outcome (Baddeley verbal reasoning task) data for the “lower cognitive score” and “higher cognitive score” groups, respectively. Usage of “Active Brains” indicated overall feasibility and satisfactory engagement with the physical activity intervention content (which did not require sustained online engagement), but engagement with online cognitive training was limited. Uptake of the additional brief telephone support appeared to be higher in the “lower cognitive score” trial. Preliminary descriptive trends in the primary outcome data might indicate a protective effect of Active Brains against cognitive decline, but further investigation in fully-powered trials is required to answer this definitively.

**Discussion:**

Whilst initial uptake and engagement with the online intervention was modest, it was in line with typical usage of other digital behavior change interventions, and early indications from the descriptive analysis of the primary outcome and behavioral data suggest that further exploration of the potential protective benefits of Active Brains are warranted. The study also identified minor modifications to procedures, particularly to improve online primary-outcome completion. Further investigation of Active Brains will now seek to determine its efficacy in protecting cognitive performance amongst adults aged 60–85 with varied levels of existing cognitive performance.

## Introduction

Dementia is a major cause of disability and dependency among older adults and places significant burden on the health and social care sector, costing US$ 1.3 trillion dollars globally in 2019 (1). Amongst adults over 60 between 12 and 18% are affected by mild cognitive impairment (MCI); (2), and up to 20% by age-associated cognitive decline (AACD); (3). Up to 10% of MCI and AACD cases progress to dementia annually (3, 4). Better preventative, diagnostic, therapeutic and social care solutions for dementia are public health priorities (1).

An estimated 40% of risk factors for dementia are modifiable, and, if managed proactively, could delay or slow disease (5). People with physically active lifestyles or those following a Mediterranean diet appear less likely to develop cognitive decline and dementia (6, 7). These behaviors also have positive effects on reducing the incidence of other risk factors including hypertension (8). Cognitive training interventions have also demonstrated potential, with reported moderate positive effects on cognitive function for healthy adults (9) and small positive effects for those with MCI (10).

The ‘FINGER' trial (*n* = 1,260) of a multidomain programme targeting diet, physical activity, cognitive training, and vascular risk monitoring, demonstrated modest reductions in cognitive decline (11). Findings suggest that addressing multiple risk factors simultaneously offers a promising strategy. However, reaching large numbers with a face-to-face delivered programme would prove prohibitively resource-intensive (12). More scalable, cost-effective models of delivering multidomain interventions are required (13).

Digital interventions may offer a feasible solution. A meta-analysis of effectiveness of web-based multidomain lifestyle programs to optimize brain health in healthy adults concluded that these interventions show an overall small-to-medium effect on outcomes (14). Whilst this offers promising evidence for the utility of digital multidomain interventions in this field, only three of the 14 identified studies in this review had evaluated interventions using controlled methods. As such there is still need for further research to provide robust Randomized Controlled Trial (RCT) evidence of effectiveness of such interventions which is the intention of the current work. Furthermore, the meta-analysis also indicated that there may be more benefit of such interventions for healthy adults compared to those with existing cognitive decline. The current work will investigate the feasibility (and ultimately effectiveness) of a multidomain intervention amongst both those with and without indications of existing cognitive decline, so could ultimately allow further exploration of whether one group may benefit more than the other. In addition, the fully powered trials of Active Brains are designed to test the effectiveness of the intervention on incidence of dementia diagnosis at 5 years as well as cognitive performance outcomes at 1 year, whereas the majority of interventions to date have only examined impact on cognitive performance. Exploration of the feasibility of Active Brains is needed to prepare appropriately for the fully-powered effectiveness evaluation.

Accordingly, we developed “Active Brains”, a multidomain web-based intervention for older adults aged 60–85 years with and without indications of existing cognitive impairment (15). Active Brains aims to reduce cognitive decline, and ultimately long-term incidence of dementia, by promoting physical activity and healthy eating behaviors, and online cognitive training. This paper presents parallel feasibility RCTs of “Active Brains” to determine the feasibility of both the intervention, and the procedures to test its efficacy and cost-effectiveness in fully powered trials.

Our main objective was to evaluate our ability to collect 80% of primary outcome data from both trial groups. The primary outcome measures were the Baddeley verbal reasoning score at 1-year follow-up, and incidence of dementia diagnosis identified from medical notes review for the proposed 5-year follow-up. The Baddeley Verbal Reasoning task was deemed the most appropriate measurement tool for the one-year primary outcome since it has been shown to be an element of cognitive functioning that is sensitive to change, and training in verbal reasoning has significant impact on the ability to maintain activities of daily living (16).

Additional objectives were to explore preliminary estimates of change in outcomes and to evaluate the feasibility and acceptability of: recruitment screening methods; trial procedures; recruitment and attrition rates (specifically the feasibility of scaling these up to the required main trial recruitment); outcome measures; engagement with the Active Brains intervention and the additional human support. We also assessed the feasibility of collecting key resource usage information, and the most suitable quality of life instruments for the full RCTs.

## Materials and methods

For further details about study design, measures and analysis, and a full description of “Active Brains” see the published protocol (17). Prospective registration of the work can be viewed here https://www.isrctn.com/ISRCTN23758980.

### Study design

We conducted parallel-design 12-month randomized controlled feasibility trials of the Active Brains intervention. Active Brains was trialed amongst two groups of adults aged 60–85 years: (1) those with indications of existing cognitive impairment (“lower cognitive score”; *n* = 180); and (2) those with no indications of existing cognitive impairment (“higher cognitive score”; *n* = 180). Trial allocation was determined by participants' baseline scores on a computerized Baddeley Verbal Reasoning task (18). The Baddeley Verbal Reasoning task offered the most suitable screening tool as it afforded the ability to draw on an extensive existing database of scores from older adults from which a normative score could be used as a meaningful threshold to base our trial allocation on. In line with existing definitions of AACD (19), a score more than one standard deviation below the “normative score” from the PROTECT database - a large (n>15,000) cohort of older adults (20) - determined allocation to the “lower cognitive score” group. The “lower cognitive score” and “higher cognitive score” groups were treated as separate trials for randomization and reporting. In each trial participants were randomized to one of three arms: (1) Active Brains; (2) Active Brains plus brief telephone/email support (telephone support as standard unless email communication preferred by participant); or (3) Usual Care comprising a single-page advice sheet about activities to protect cognitive health.

### Study setting and recruitment

Between October 2018 and January 2019, 19 primary care practices in Hampshire, Dorset and Wiltshire completed a database search, screen and mailout in accordance with the study's eligibility criteria. Participating practices recorded age, gender, and postcode of all invitees. Mailout packs provided immediate access to the Active Brains website where invitees could: sign up, provide informed consent, complete additional screening (including the Baddeley Verbal Reasoning Task to determine trial assignment) and, if eligible, complete baseline measures and randomization. Anyone whose score allocated them to the “higher cognitive score” group after 180 participants had been allocated entered a non-randomized “cohort” group with access to Active Brains. This paper focuses only on the RCT participants.

### Measures and data collection procedures

#### Baseline

At baseline, all measures were completed online after initial sign-up and online consent. The cognitive assessment tasks (comprising the Baddeley Verbal Reasoning task, the digit span task, the paired associates learning task and the self-ordered search task) were accessed *via* the PROTECT platform (21). The cognitive assessment tasks have been running for seven years on the PROTECT platform and prior to this have a 35-year history of use. The data flow and management of the cognitive tests is monitored by a cognitive testing expert. The cognitive assessment tasks delivered *via* the PROTECT platform and used in the Active Brains study are well established as a cognitive assessment tool. Several very large studies have demonstrated their validity (21–23). A description of these four cognitive assessment tasks can be found in a prior publication from the PROTECT study team [(22); p992]. In the Baddeley Verbal Reasoning task participants had 3 min to respond to as many statements as possible which referred to the relative size and positional relationship between a changing image of a square and a circle. Examples of these statements include “The square is bigger than the circle,” “The circle does not contain the square.” These included a mixture of positively or negatively worded, and true or false statements. Participants indicated whether the statement was true or false by clicking on the appropriate button on the page. Correct responses added to their total score, whereas incorrect responses deducted from it. All other measures were completed *via* the Active Brains website by those eligible.

#### 12-month follow-up

All participants were invited *via* email at 1-year post-randomization to complete online follow-up measures. Participants who had not completed the online cognitive assessment tasks, the Instrumental Activities of Daily Living (IADL); (24) and the EQ5D-5L (25) measures after 3 weeks (including two additional reminder emails) were contacted by paper mail-out and, where necessary, by phone to prompt completion of measures. The cognitive assessment tasks (comprising the Baddeley Verbal Reasoning task, the digit span task, the paired associates learning task and the self-ordered search task) could only be completed online. If these measures remained incomplete after 8 weeks with no participant contact, emails and phone calls were made to the participant's nominated contact person if provided. These requested that the contact person prompted or supported the participant to complete the measures or, if not possible, for the contact person to complete the Informant Questionnaire on Cognitive Decline in the Elderly (IQCODE); (26) and proxy versions of the IADL and EQ-5D.

#### Notes review

A medical notes review data collection form and accompanying instructions were shared with recruiting primary care practices. These asked practice staff to report for each of their randomized patients: major medical conditions diagnosed before and during the study period; family history of dementia; any record of cognitive impairment complaints; blood pressure and cholesterol readings; medications prescribed at baseline and medication changes during the study period; and healthcare use including primary care consultation, outpatient attendance, A & E visits, and hospitalization during the study period. Should participants have moved and changed practices during the study period, the study team planned to still collect primary care data if they moved to another participating practice. If they moved outside of the participating regions or to a non-participating or non-research active practice, collection of primary care data would not have been possible for this individual.

### Analysis

The completeness of the primary outcome data and the other key feasibility outcomes including: intervention uptake, adherence, attrition, retention, and the number of participants recruited per practice, healthcare costs and quality of life were summarized descriptively. Baseline participant characteristics and trial outcomes were descriptively analyzed as randomized (i.e., regardless of level of engagement with the intervention), using number (%), mean (SD) or median (IQR) as appropriate. The pattern and frequency of missing data was also descriptively explored. The completeness of primary outcome and notes review data were used to answer the primary research question about whether we could collect 80% of our outcome data. The recruitment data (uptake rate, total number recruited, recruits per practice and recruitment duration) were used to determine the feasibility of scaling up our recruitment to reach the required sample sizes for the main trials.

## Results

From 5,475 study invitations, 1,001 individuals (18.3%) completed online sign-up. Amongst those choosing not to participate, 18.5% (*n* = 828) returned a reply slip indicating why. The most common reasons related to lack of access, or unwillingness to use computers or the internet, or commitments including work and caring responsibilities.

Of 1,001 individuals who signed up, 2% (*n* = 21) did not complete consent, and a further 7% (*n* = 70) did not complete the online screening, leaving 910 individuals who were assessed for eligibility. Overall, 10.2% of invitees (*n* = 560) participated.

**Figure 1:**
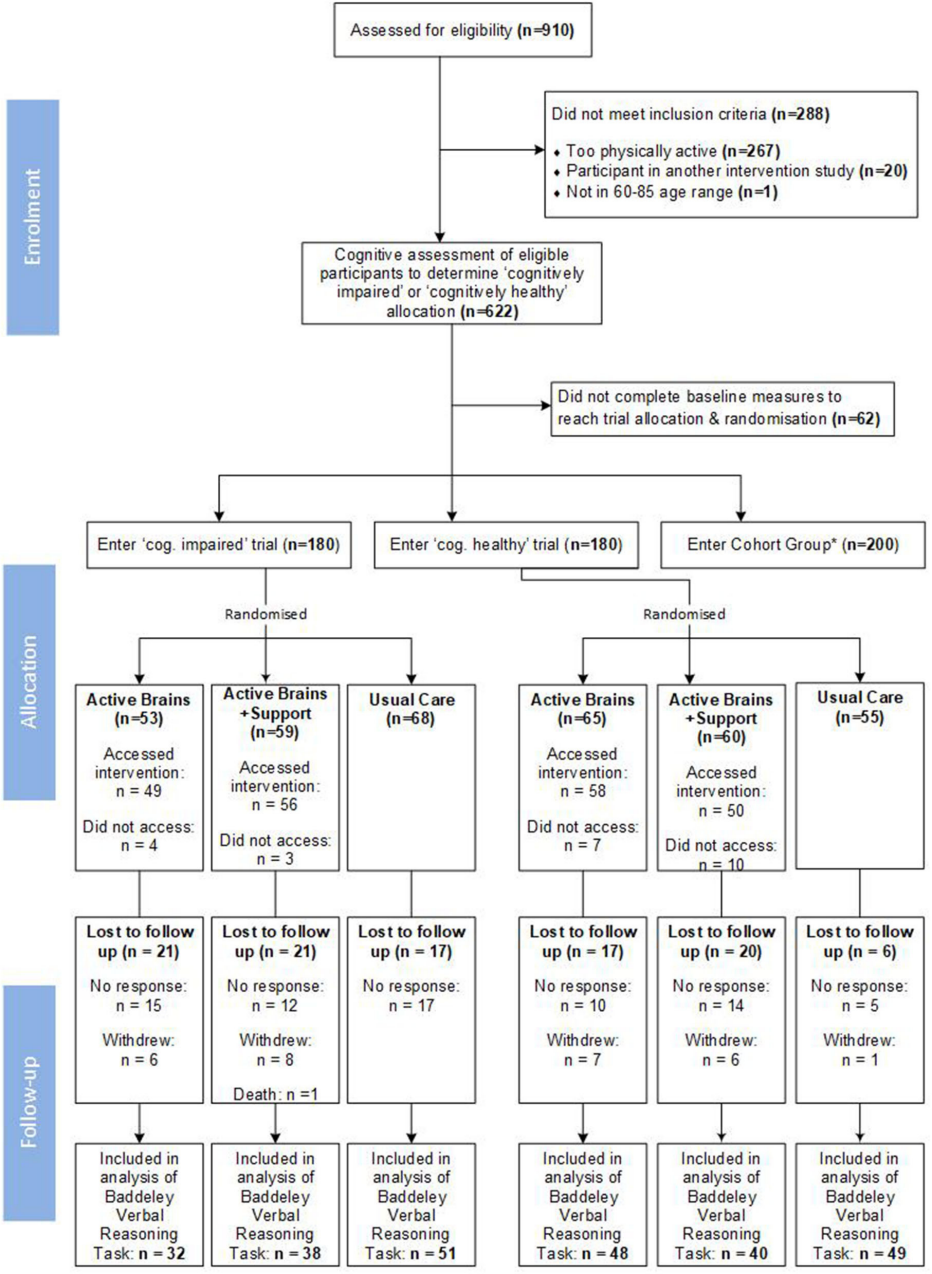
Recruitment and retention into the parallel trials; *Cohort group data not presented within this paper.

Figure 1 illustrates participant flow through the trials.

### Sample characteristics

Participant characteristics for each group (Table 1) were generally evenly distributed in each trial. Participants in the “higher cognitive score” trial were slightly younger, with a larger proportion having Higher Education qualifications, compared to those in the “lower cognitive score” trial. Samples were predominantly White (British, Irish or European), living with a partner and regular users of the internet.

**Table 1:** Participant baseline characteristics in each trial.

	**“Lower cognitive score” trial**			**“Higher cognitive score” trial**		
	**Active Brains** ***n =*** **53**	**AB + support** ***n =*** **59**	**Usual care** ***n =*** **68**	**Active Brains** ***n =*** **65**	**AB + support** ***n =*** **60**	**Usual care** ***n =*** **55**
**Female**	34 (64.2%)	33 (55.9%)	43 (63.2%)	25 (38.5%)	32 (53.3%)	34 (61.8%)
**Age (mean, SD)**	71.1 (6.8)	70.9 (5.5)	70.8 (5.7)	67.9 (5.3)	67.8 (6.0)	67.5 (5.1)
**Living situation**						
On my own	10 (18.9%)	12 (20.3%)	25 (36.8%)	9 (13.8%)	13 (21.7%)	9 (16.4%)
With a spouse/partner	40 (75.5%)	45 (76.3%)	39 (57.4%)	54 (83.1%)	45 (75.0%)	45 (81.8%)
With a relative	3 (5.7%)	2 (3.4%)	4 (5.9%)	2 (3.1%)	1 (1.7%)	1 (1.8%)
With a friend	0 (0.0%)	0 (0.0%)	0 (0.0%)	0 (0.0%)	1 (1.7%)	0 (0.0%)
**Ethnic group**						
White	53 (100.0%)	58 (98.3%)	68 (100.0%)	64 (98.5%)	59 (98.3%)	55 (100.0%)
Mixed	0 (0.0%)	0 (0.0%)	0 (0.0%)	1 (1.5%)	1 (1.7%)	0 (0.0%)
Asian	0 (0.0%)	0 (0.0%)	0 (0.0%)	0 (0.0%)	0 (0.0%)	0 (0.0%)
Black	0 (0.0%)	0 (0.0%)	0 (0.0%)	0 (0.0%)	0 (0.0%)	0 (0.0%)
Other	0 (0.0%)	1 (1.7%)	0 (0.0%)	0 (0.0%)	0 (0.0%)	0 (0.0%)
**Education level**						
Secondary	20 (37.7%)	22 (37.3%)	27 (39.7%)	11 (16.9%)	11 (18.3%)	15 (27.3%)
Post-secondary	9 (17.0%)	9 (15.3%)	10 (14.7%)	10 (15.4%)	11 (18.3%)	9 (16.4%)
Vocational	16 (30.2%)	12 (20.3%)	16 (23.5%)	17 (26.2%)	13 (21.7%)	14 (25.5%)
Undergraduate	6 (11.3%)	11 (18.6%)	10 (14.7%)	17 (26.2%)	17 (28.3%)	13 (23.6%)
Post-graduate	1 (1.9%)	3 (5.1%)	4 (5.9%)	6 (9.2%)	6 (10.0%)	3 (5.5%)
Doctorate	1 (1.9%)	2 (3.4%)	1 (1.5%)	4 (6.2%)	2 (3.3%)	1 (1.8%)
**Internet use**						
Every day	43 (81.1%)	40 (67.8%)	51 (75.0%)	55 (84.6%)	54 (90.0%)	46 (83.6%)
A few times each week	10 (18.9%)	19 (32.2%)	15 (22.1%)	10 (15.4%)	6 (10.0%)	8 (14.5%)
A few times a month	0 (0.0%)	0 (0.0%)	1 (1.5%)	0 (0.0%)	0 (0.0%)	1 (1.8%)
Less often	0 (0.0%)	0 (0.0%)	1 (1.5%)	0 (0.0%)	0 (0.0%)	0 (0.0%)

Participants were comparable to non-participants in terms of mean age (69.2 years vs. 69.9 years, respectively), demonstrated a similar gender balance (51.7% of participants vs. 49% invited were female), but areas of lower relative deprivation were over-represented (mean index of multiple deprivation (IMD) was 7.5 in participant group vs. 6.8 in non-participants); (27). Indices of Multiple Deprivation (IMD) are the official measure of relative deprivation in England. They collate weighted data from across seven domains of deprivation for each neighborhood in the country to create a rank of most to least deprived areas. IMD is often expressed in deciles, with the top 10% (most deprived areas in the country) being in the first decile (IMD = 1), and the bottom 10% (least deprived areas in the country) being in the tenth decile (IMD = 10) (28).

The remainder of the results section reports our findings relating to the acceptability and feasibility of: (1) the Active Brains intervention, and (2) the proposed trial procedures. The study was not powered to perform statistical comparisons. In line with the CONSORT 2010 statement extension to feasibility and pilot studies (29) all reporting of results refers to descriptive statistics and narrative description of apparent trends in the data from visual inspection only. Some of the reporting comments on ostensible differences in the descriptive data over time or between groups. However, we acknowledge that these do not necessarily reflect statistically significant differences at this point and are simply indications of what may be important to explore in fully powered analyses.

### Acceptability and feasibility of “Active Brains” – Initial uptake, usage and preliminary indications of change

#### Initial uptake and usage of active brains

Table 2 below briefly outlines the modules within Active Brains and when these became available to participants in the intervention arms of the trials.

**Table 2:** Active Brains modules and release schedule.

**Active Brains module**		**Description**	**Available**
Active Lives	Getting active	Advice and support for increasing general physical activity levels	Immediately – as soon as participant is randomized to Active Brains or Active Brains plus support group
	Strength and balance	Support for building strength and balance exercises into daily routines	
	Breaks from sitting	Advice and support for breaking up sedentary time	
Brain training		Advice about keeping the brain active and access to brain training games [described here (22); Table 1] with advice to aim for 3–5 sessions of brain training per week for first 6 months	4 weeks after randomization – 6 Brain training games available immediately, 1 additional game added every 4 weeks up to maximum of 12 games
Eat for Health		Advice and support for modifying eating patterns toward a healthier diet, with a particular focus on elements of a Mediterranean style diet (e.g., nuts, leafy green veg, oily fish)	8 weeks after randomization
Active Brains “Booster” content		Access to all existing modules, plus some modified/additional content: - Recommendations to carry out Brain Training 3–5 times a week for 1 month, every 3 months - Additional links to recipes/resources for meal planning aligned to healthy eating recommendations - Additional links to external resources for finding activities within community/local area.	7 months after randomization

Initial uptake (accessing Active Brains at least once) of the intervention amongst those in the intervention arms (with or without support) appeared to be higher in the “lower cognitive score” trial at 93.8%, compared to 86.4% in the “higher cognitive score” trial. Usage of Active Brains is shown in Table 3.

**Table 3:** Usage of Active Brains components by trial and trial arm.

	**% of people (and number) from each workstream and group who accessed at least once**					
**Section**	**Lower cognitive score (*****n*** **= 112)**		**Higher cognitive score (*****n*** **= 125)**		**Combined (*****n*** **= 237)**	
	AB (*n* = 53)	AB + S (*n* = 59)	AB (*n* = 65)	AB + S (*n* = 60)	AB (*n* = 118)	AB + S (*n* = 119)
Overall uptake (any use)	92.5% (49)	94.9% (56)	89.2% (58)	83.3% (50)	90.7% (107)	89.1% (106)
Active Brains introduction	92.5% (49)	93.2% (55)	87.7% (57)	83.3% (50)	89.8% (106)	88.2% (105)
Active Lives (AL) intro	84.9 % (45)	84.7% (50)	78.5% (51)	75% (45)	81.4% (96)	79.8% (95)
AL: Getting active	56.6% (30)	57.6% (34)	61.5% (40)	61.7% (37)	59.3% (70)	59.7% (71)
AL: Strength and balance	49.1% (26)	61% (36)	46.2% (30)	50% (30)	47.5% (56)	55.5% (66)
AL: Breaks from sitting	32.1% (17)	45.8% (27)	40% (26)	35% (21)	36.4% (43)	40.3% (48)
Brain Training (BT)^a^	58.5% (31)	72.9% (43)	58.5% (38)	65% (39)	58.5% (69)	68.9% (82)
Number of BT sessions^b^	848	838	1,113	1,062	1,961	1,900
Median BT sess. per user	3	5	3	5	3	5
Participants meeting BT recommendations^c^	3.8% (2)	0	4.6% (3)	1.7% (1)	4.2% (5)	0.8% (1)
Eat for Health	52.8% (28)	47.5% (28)	46.2% (30)	53.3% (32)	49.2% (58)	50.4% (60)

a Brain Training (BT), proportion (number) of participants who accessed the Brain Training module;

b BT sessions, number of separate occasions Brain Training games were accessed across all individuals in this group;

c Brain Training module recommended that participants accessed BT games 3–5 times per week for first 6 months.

Across both trials, and regardless of support provision, there was relatively high initial use of Active Brains online content followed by a gradual decline over time. The three main components of Active Brains are released sequentially (Active Lives immediately, Brain Training after 4 weeks, Eat for Health after 8 weeks), and ~50% of participants were still accessing Active Brains once all content was available. “Getting Active” was the most accessed of the (simultaneously available) physical activity sub-modules. Access to “Breaks from Sitting” (reducing sedentary time) was relatively low across all users but was perhaps slightly higher in the supported group in the “lower cognitive score” trial.

Larger proportions of the supported groups seemed to access the Strength and Balance and Brain Training modules - especially in the “lower cognitive score” trial. Whilst the frequency of use of Brain Training games per participant was low overall, those in the supported groups appeared to access the games more frequently – either with larger proportions of the supported group seeming to access the games (in the “lower cognitive score” trial), or the individuals in the supported group who were accessing the games appearing to do so more frequently (in both trials). However, very few participants in either trial demonstrated the recommended level of use of accessing the Brain Training games 3–5 times per week for the first 6 months. Use of “Eat for Health” was consistent across groups, with ~50% of users accessing this.

#### Initial uptake of, and adherence to, human support

Participants randomized to the support groups were offered up to three brief telephone support calls (or email support if they preferred) with a trained facilitator during the first 12 weeks. These appointments only occurred if the participant contacted their designated supporter to arrange them. In addition, all support arm participants received automated emails from their designated supporter at 3- and 7-weeks post-randomization. These provided general encouragement and a reminder of the further support available. Engagement with these support opportunities is shown in Table 4.

**Table 4:** Uptake and use of support provision.

**Support type**	**Lower cognitive score support arm (*n* = 59)**	**Higher cognitive score support arm** **(*n* = 60)**	**Combined support arm across both trials (*n* = 119)**
**Participant made email contact**	68% (40)	53% (32)	**61% (72)**
**At least one phone appointment**	54% (32)	47% (28)	**50% (60)**
**At least two phone appointments**	27% (16)	13% (8)	**20% (24)**
**All three phone appointments**	7% (4)	0	**3% (4)**
**Requested additional phone appointment**	2% (1)	0	**1% (1)**

More than half of all support arm participants contacted their supporter, and half had at least one telephone support appointment. Initial uptake of support (i.e., any engagement with the designated supporter) appeared to be higher in the “lower cognitive score” trial. Those in this trial also seemed more likely to request multiple appointments. Feedback from supporters was collated *via* email and group discussion and is reported in Supplementary Table 1.

#### Preliminary estimates of change

##### Primary outcome data

Although the feasibility trials were not powered to make statistical comparisons between groups, we can comment briefly on possible indicative patterns in the descriptive analysis of primary outcome data (Table 5).

**Table 5:** Baddeley Verbal Reasoning task scores and number meeting AACD/MCI criteria.

**Lower cognitive score Trial**	**Active Brains (*****n*** = **53)**		**Active Brains** + **support (*****n*** = **59)**		**Usual care (*****n*** = **68)**	
	**Baseline**	**Follow up**	**Baseline**	**Follow up**	**Baseline**	**Follow up**
**Baddeley Verbal Reasoning score** Mean (SD)	16.4 (4.9)	18.7 (6.6)	15.2 (5.8)	19.1 (8.7)	14.7 (5.8)	19.45 (7.3)
**AACD flag** *n* (%) (1 SD below norm verbal reasoning)	53 (100%)	23/32 (71.9%)	59 (100%)	25/38 (65.8%)	68 (100%)	39/51 (76.5%)
**MCI flag** (1.5 SD below norm verbal reasoning)	27(50.9%)	13/32 (40.6%)	35 (59.3%)	15/38 (39.5%)	45 (66.2%)	20/51 (39.2%)
Missing score (*n*, %)	0 (0.0%)	21 (39.6%)	0 (0.0%)	21 (37.3%)	0 (0.0%)	17 (25.0%)
**Higher cognitive score Trial**	**Active Brains (*****n** =* **65)**		**Active Brains** + **support (*****n** =* **60)**		**Usual care (*****n** =* **55)**	
	**Baseline**	**Follow up**	**Baseline**	**Follow up**	**Baseline**	**Follow up**
**Baddeley Verbal Reasoning score** Mean (SD)	31.4 (6.2)	32.1 (8.1)	31.6 (6.7)	32.2 (8.1)	30.4 (4.9)	28.8 (7.8)
**AACD flag** (1 SD below norm verbal reasoning)	0 (0.0%)	5/48 (10.4%)	0 (0.0%)	5/40 (12.5%)	0 (0.0%)	12/49 (24.5%)
**MCI flag** (1.5 SD below norm verbal reasoning)	0 (0.0%)	1/48 (2.1%)	0 (0.0%)	1/40 (2.5%)	0 (0.0%)	5/49 (10.2%)
Missing score (*n*, %)	0 (0.0%)	17 (26.2%)	0 (0.0%)	20 (33.3%)	0 (0.0%)	6 (10.9%)

In the “lower cognitive score” trial all trial arms appeared to show higher mean verbal reasoning scores at follow-up, as well as seemingly smaller proportions of respondents meeting AACD/MCI criteria. This potential change in proportion of respondents meeting the AACD criteria appears more pronounced in intervention arms compared to usual care.

In the “higher cognitive score” trial, verbal reasoning scores appeared to remain consistent between baseline and follow-up in the two intervention arms and possibly showed a small decline in the usual care group. Relatedly, the proportion of participants meeting the AACD/MCI criteria at follow-up appeared to increase more sharply in the usual care arm compared to the intervention arms.

An imputed analysis, including auxiliary variables and predictors of missing AACD outcome, gave similar estimates to the observed proportions. This assumes that missing outcomes are missing at random given the observed data.

##### Intervention-targeted behaviors

Reviewing the descriptive analysis of data relating to behaviors targeted by the intervention gives insight into trends to explore further in fully powered trials. Supplementary Tables 2, 3 report the brain training-related behaviors outside of Active Brains and healthy eating data, respectively, which gave little indication of potential change or difference between groups. However, reviewing the physical activity descriptive data (Table 6) indicates some trends that may be important to explore further.

**Table 6:** Baseline and follow-up IPAQ-E (physical activity) data in each trial.

**Lower cognitive score Trial**	**Active (*****n** =* **53)**		**AB** + **Support (*****n** =* **59)**		**Usual care (*****n** =* **68)**	
	**Baseline**	**Follow up**	**Baseline**	**Follow up**	**Baseline**	**Follow up**
IPAQ mean (SD) MET min per week	4,238.2 (2,670.2)	5,414.9 (3,475.6)	3,459.8 (2,551.9)	3,656.2 (3,121.8)	4,302.7 (2,709.3)	4,577.4 (2,676.2)
Mean (SD) min per week walking	753.5 (423.3)	825.1(444.8)	604.9(372.0)	617.7 (395.7)	625.8 (389.9)	671.2 (411.0)
Mean (SD) min per week moderate	463.8 (371.0)	632.7(441.4)	379.1(265.6)	429.0 (346.3)	465.9 (335.6)	461.6 (347.7)
Mean (SD) min per week vigorous	137.8 (110.7)	217.0(162.9)	206.6(166.5)	213.8 (166.2)	220.3 (185.7)	254.5 (201.5)
Mean (SD) min per week strength/balance^a^	93.6 (54.7)	247.5(306.0)	107.6 (99.5)	118.6 (148.4)	149.3 (175.4)	149.5 (118.4)
Missing IPAQ (*n*, %)	0 (0.0%)	25 (47.2%)	0 (0.0%)	29 (49.2%)	0 (0.0%)	20 (29.4%)
**Higher cognitive score Trial**	**Active Brains (*****n** =* **65)**		**AB** + **Support (*****n** =* **60)**		**Usual care (*****n** =* **55)**	
	**Baseline**	**Follow up**	**Baseline**	**Follow up**	**Baseline**	**Follow up**
IPAQ mean (SD) MET min per week	3,362.2 (1,853.2)	4,157.0 (2,944.8)	3,488.3 (2,457.5)	3,607.2 (2,052.9)	4,449.2 (2,647.5)	4,004.1 (2,413.7)
Mean (SD) min per week walking	625.1 (389.6)	673.0(393.0)	612.0(401.5)	588.3 (383.0)	694.1 (432.8)	647.7 (401.3)
Mean (SD) min per week moderate	302.7 (238.3)	396.0(310.4)	339.4(327.5)	366.3 (309.0)	537.3 (415.2)	390.6 (313.8)
Mean (SD) min per week vigorous	155.6 (141.4)	249.3(308.2)	96.7 (65.5)	94.3 (71.2)	118.2 (80.4)	194.7 (160.6)
Mean (SD) min per week strength/balance^a^	93.5 (88.5)	127.4(113.7)	72.1 (43.6)	123.5 (137.6)	91.4 (54.3)	101.6 (78.7)
Missing IPAQ (*n*, %)	0 (0.0%)	20 (30.8%)	0 (0.0%)	20 (33.3%)	0 (0.0%)	9 (16.4%)

a An item relating to frequency and duration of strength and balance was added for the present study in the format of the IPAQ questionnaire but does not typically feature in the validated version of this survey.

The International Physical Activity Questionnaire (IPAQ-E); (30) data indicated high levels of physical activity amongst participants in both trials at baseline. At follow-up, in the “lower cognitive score” trial there seemed to be a more apparent increase in all domains of activity in the Active Brains only group compared to either the supported group or usual care. In the “higher cognitive score” trial there was a similar, but less pronounced pattern. Here the Active Brains group appeared to show smaller increases across physical activity domains than in the “cognitively impaired” trial, but still seemingly larger than in the support and usual care groups.

Uptake of a free pedometer from Active Brains appeared to be slightly higher in the “lower cognitive score” trial (35%, *n* = 63) compared to the “higher cognitive score” trial (29%, *n* = 52).

### Acceptability and feasibility of trial procedures

#### Collection of primary outcome data

##### Online Baddeley verbal reasoning task

Completion of the Baddeley verbal reasoning data was 76.1% in the “higher cognitive score” trial and 67.2% in the “lower cognitive score” trial. Completion of *some* outcome data regardless of whether the online primary outcome was completed, was 80% in both trials.

In both trials, the highest completion of the primary outcome was in the usual care groups. Supplementary Table 4 provides a breakdown of primary outcome completion by trial and intervention group.

As our primary outcome completion fell short of the 80% target in both trials, we took measures to address identified issues (Table 7).

**Table 7:** Issues with primary outcome collection and mitigating measures.

**Problem identified**	**Mitigation measures(s) implemented**
Less than 80% completion of online primary outcome task – especially amongst participants in “lower cognitive score” trial.	• Reordered follow-up process: moved phone call ahead of sending paper measures (to avoid completion of paper measures without primary outcome)
	• Changed focus of call to prompting/supporting participants to complete online measures (by focusing the call on asking people if we could talk them through accessing the online tasks whilst we were on the phone to them, or providing additional instruction verbally or *via* email, instead of trying to collect other measures over the phone as previously planned)
	• Added step to follow-up process between automated emails and phone call: postal reminder to complete follow-up online with detailed instructions about how to access and complete online tasks. This sought to give participants who may have been unsure about how to access the online tasks additional written instructions they could refer to that were not part of originally planned procedures.
Message possibly not clear that online tasks were the most important element and could be completed quickly	• Changes to PIS documents and automated email prompts to emphasize importance of completing online task even if no time for other parts
	• Provided a time estimate for completion of tasks (10 min) to illustrate they could be completed quickly
	• Adaptation of paper follow-up packs splitting into very brief “primary outcomes” (IQCODE, IADL and EQ5D), plus additional longer pack of secondary measures. Accompanying letter stresses importance of online tasks, and signposts to this.
Possibility of automated emails being ignored/spam filtered so people miss request to complete online measures	• Amended emails to be sent from named email account; more clearly distinguishes them from other emails from Active Brains that don't require action
Higher withdrawal rate from intervention groups limiting maximum possible follow-up	• Clarified existing “partial withdrawal” (for participants wanting to cease use of Active Brains/ receiving intervention emails, but happy to be contacted to complete follow-up measures) option: made it clear to participants they can choose “partial withdrawal” even if only happy to complete the online primary outcome task.
	• Amended online system to allow participants more control over self- selecting level of withdrawal. Aim to maximize partial (primary) outcome data from those who would otherwise provide none.

##### Notes review

We collected notes review data for 94.4% of participants across both trials. Data were analyzed to inform any amendments required to the notes review data collection form and choice of instruments to measure quality of life. Detailed collection of medication use both at baseline and follow up proved too complex and time-intensive. We simplified the data form by collecting information only if any medication changes occurred during the study. Additional data about health service resource use is provided in Supplementary Table 5.

We explored use of three quality life and well-being measures –EQ5D-5L, the SF-12 (31) and the Index of Capability for Older Adults [ICECAP-O; (32)]; response rates were similar at both baseline and 1-year follow-up. EQ5D-5L and SF12 were more sensitive to QoL variation compared with ICECAP-O and feedback from Patient and Public Involvement Contributors (PPI) that ICECAP-O's items may be difficult or off-putting to answer led us to removing this measure. The EQ-5D-5L and SF-12 were deemed sufficient to capture quality of life and well-being data in the trial population.

#### Evaluating trial procedures

We evaluated the acceptability of the study's: screening methods, recruitment strategies, randomization process, study materials, outcome measures, notes review process, and recruitment and attrition rates. Our screening methods appeared largely feasible to operationalize and were effective in recruiting eligible samples for each trial. Throughout the screening process, several minor issues were identified and addressed as described in Supplementary Table 6.

Our key findings regarding trial procedures and the associated implications for the main trial are shown in Table 8.

**Table 8:** Acceptability of study processes - key findings and implications for trial.

**Key findings**	**Implications for main trial**
**Recruitment strategies**
• Primary care recruitment proved feasible to recruit to target (*n* = 360) within timeframe (3 months)	Primary care recruitment only feasible
	Based on need to recruit *n* = 21,455 across both trials, will need to recruit approx. 740 GP practices
• Practices reported database search criteria easy to operationalise	
• Practices identified screening process as the most resource intensive aspect, and determinant of maximum mailout size.	
• Average mailout size = 288	
• Average number of participants recruited per practice = 29	
• Alternative routes explored (e.g., Join Dementia Research, Dementia Platforms UK, poster recruitment) not feasible for various reasons: º no clear pathway to access participants' medical notes for review at the end of the study; º administrative procedures required far too resource intensive at the scale required; º screening out ineligible invitees not possible/easy	
**Randomization**
• Pure randomization	Randomization method will be taken forward to main trial unchanged
• Performed automatically ‘behind the scenes' by the Active Brains website, allowing participants seamless transition from baseline measures to notification of group allocation • Resulted in relatively evenly balanced groups even in small sample	
• No reported issues with randomization	
**Study materials**
• Participant facing materials and instructional documents for GP practices generally accessible and easy to follow	Modifications to participant facing documentation detailed in Table 6
• Clarifications required to information sheet/ follow-up questionnaires/ cover letter to maximize completion of online primary outcome	
**Outcome measures**
• Overall good completion – 80% in both trials provided some follow-up data	Modifications to participant facing documentation detailed in Table 6 to facilitate improved completion of primary outcome Continued use of IPAQ-E to measure physical activity. Minor modifications to IADL measure.
• Little missing data due to online completion automatically flagging missed responses	
• Slightly lower than hoped completion of primary outcome – particularly in “lower cognitive score” trial	
• Some issues identified with IPAQ-E and IADL measures	
**Notes review**
• Highly successful in collecting required data – 94.4% of whole sample notes review data collection; 100% of the data requested	Removed baseline medications and medical conditions questions and only record any changes/ additions since baseline. Added questions on these items to participants' baseline measures.
• Instructional document ensured form was easy to complete, but practice staff expressed concerns that it was too time consuming per patient, especially re. medical conditions and medications.	
**Recruitment and attrition rates**
• 10.2% randomization rate – 560 participants from 5,475 invites	Confirms need for 730–740 GP practices.
• 92.2% retention rate – 28 withdrew, 14 from each trial	Amendments to ensure: 1) clear to participants they could withdraw from use of the intervention without leaving the trial; and 2) participants could more easily self-action withdrawal *via* the website.
• Across both trials, withdrawal rate from intervention substantially higher than Usual Care - 0.8%; Active Brains - 10.2%, Active Brains Plus Support - 12.6%	
• Loss to follow-up (i.e., no completion of primary outcome amongst those who remained in the study) higher in “lower cognitive score” trial (24.4%) than in “higher cognitive score” trial (16.1%)	Changes to follow-up materials to facilitate completion of primary outcome online documented in Table 6.

##### Minor changes to measures

We identified potential issues with two measures; the IPAQ-E and the IADL.

##### IPAQ-E

The IPAQ-E scores indicated very high baseline levels of physical activity across all groups. Given our exclusion of highly active individuals [using the Godin Leisure Time Exercise Questionnaire (33)], this was unexpected, leading to concerns about the IPAQ-E's validity. Despite considering potential alternatives, the IPAQ-E was still considered the most accessible self-report instrument with a level of granularity that should permit detection of small changes. This is pertinent given that Active Brains advocates small, gradual change in physical activity behavior.

##### IADL

Through feedback from PPI, participants, and team discussion, we identified some potential issues with the wording of the IADL items. Each item in the IADL has two parts asking the respondent to report: (a) how much assistance they have with that specific activity, (b) and how difficult they find the activity. The first part of the question did not appear to distinguish between activities that were not performed because the individual was not capable of performing it and those that were not performed because the activity was not relevant to the individual (i.e., because it was not an activity they need to do - e.g., taking medications, or not one that they took responsibility for within their household - e.g., managing finances). Furthermore, the second part of the question asked the participant to report how difficult they found the activity, even if they had previously reported that the activity was not performed or was done with full assistance which respondents reported finding confusing. Accordingly, we made some minor wording and formatting changes to these questions so that respondents could: (1) indicate whether they were unable to complete an activity themselves or if that activity was not relevant to them, and (2) indicate how much difficultly they had or would have with conducting the activity – even if not one they complete themselves.

To check that our modifications did not systematically affect participant responding on the IADL, we only modified the paper version sent to those who did not complete measures online. This allowed comparison between the data collected with the original version and our modified version. The distributions of the two data sets were broadly similar indicating no cause for concern, so these changes were applied to the online version.

## Discussion

This study provides insight into engagement with the Active Brains intervention; provides preliminary interpretations of ostensible trends in outcomes at 1 year and evaluates the feasibility and acceptability of study procedures. These investigations were conducted amongst adults aged 60–85 with, and without, indications of existing AACD or MCI. The findings are important for determining the feasibility of planned future work to evaluate Active Brains.

### Uptake and engagement with active brains

Initial uptake of the study invitation (18.3%) was in line with expectations for a UK primary care mail-out study to older adults even without an online aspect to the study (34, 35). Although a commonly reported reason for non-participation was lack of access to, or willingness to engage with the internet, only 15% of those invited reported a reason for non-participation, and many selected other reasons alongside these such as working or caring responsibilities. As such, this did not raise undue concerns about the potential future application and accessibility of Active Brains amongst UK older adults. In the 7 years between 2013 and 2020, internet use amongst over 75s in the UK nearly doubled from 29% to 54% (36). Older adults are the most rapidly growing users of the internet, and whilst there is inevitably some within this age group who do not currently use the internet, it is likely that this will continue to rapidly decline. As time goes on, we believe that digital interventions such as Active Brains will become increasingly accessible amongst large proportions of UK older adults.

Whilst the Active Brains usage data indicated that use of the online components was fairly modest, there was still evidence of the intervention being feasible and acceptable for participants in both trials to access and use, with 50% still accessing it at 2 months. Such levels of online usage are very much in line with that of other web-based health behavior change interventions (37). Furthermore, the online usage statistics alone do not necessarily reflect all engagement with the recommendations of the intervention, as discussed further below. Exploring the impact of engagement with intervention content, and adherence to intervention recommendations will be a key part of the process evaluations conducted alongside the main trials, as recommended by recent guidance (38). If the slightly higher proportion of “lower cognitive score” participants accessing Active Brains overall (94% vs. 86% of the “higher cognitive score” participants) reflects a real difference, this may indicate a greater perceived relevance of the intervention amongst this group. Previous research has demonstrated that self-perceived cognitive deficit predicts willingness to invest time in interventions to protect cognition (39).

Usage of the online brain training element of Active Brains was low and, amongst nearly all participants in both trials, did not reflect the intervention's recommendations (3–5 times per week for an initial 6-month period). Across all groups, the median number of brain training sessions per user indicates lower usage than in a previous trial of the same cognitive training tasks that demonstrated a significant benefit for older adults' cognitive function (22). Our qualitative process data (to be reported elsewhere) indicated that many participants got bored of the games quite quickly which may explain low continued engagement. This may partially be explained by a programming error early in the feasibility trials which meant that the intended release schedule of the games (i.e., an initial six games with one additional game every 4 weeks up to a total of 12 games) was sped up meaning that all games became available to users within a much shorter period of time. This has been resolved for the main trials and so may facilitate more prolonged engagement with the novelty of available games lasting longer. Whilst sufficient engagement with the brain training games is important, exactly what “sufficient engagement” is in the context of a multi-domain intervention such as Active Brains is complex. For example, participants may have only accessed components they felt they needed support with. Recent evidence from a study examining dose-response in a multi-domain dementia prevention intervention suggests that higher number of sessions engaged with was not necessarily optimal for cognitive outcomes (40). Active Brains may also have prompted users to engage in other brain training activities – i.e., other online games, or pursuing “offline” activities.

Despite this lower than anticipated engagement with the online brain training, the behavioral data gives very preliminary indications that some aspects of the physical activity recommendations may have been better engaged with – more likely those from the Getting Active and Strength and Balance sub-modules given that these appeared to be more widely engaged with. Although recognized that the IPAQ-E can over-estimate time spent across all activity intensities and underestimate sedentary time amongst older adults (41), inflation of physical activity estimates here are likely to be present at both baseline and follow-up and so higher scores at follow-up could still be indicative of actual change. The IPAQ-E data seemed to indicate possible increases in physical activity behavior in the Active Brains group – particularly in the “lower cognitive score” trial. Active Brains was developed with the intention of minimizing users' need to regularly access online content and to instead build activity into daily routine and habits. Accordingly, sustained online engagement with the online content prompting physical activity was not considered necessary to support “effective engagement” (42) with the intervention. The suggestion of a possible increase in physical activity in the Active Brains groups is promising given that recent syntheses of the evidence about modifiable dementia risk factors indicate that interventions to enhance physical activity behavior point toward small beneficial effects for cognition overall, whereas those for cognitive training are somewhat less conclusive (5). If reflective of a significant statistical difference in a larger sample, the possible larger IPAQ-E increases in the Active Brains only groups compared to the supported groups could indicate that brief human support may not be beneficial (or may even be detrimental) in relation to independently sustaining physical activity behavior. These speculations can be further explored in the fully powered trials.

About half of those offered additional support in each trial had at least one telephone appointment, with larger numbers making at least email contact with their supporter. This is comparable to support uptake amongst digital behavior change interventions with similar models of brief additional support (43, 44) and even to those that have demonstrated better intervention usage and trends toward better outcomes with only modest uptake of support (45). It possible that, amongst those who did not actively engage with their supporter, email reminders of the availability of support calls should they want them may have offered a sufficient level of perceived support. Indeed, our qualitative process data (to be published elsewhere) also indicated that even amongst those who did not take up the offer of support, they found it useful to know it was there if they needed it. In terms of the feasibility of scaling up support provision for the main trials, brief telephone support will continue to be provided by centralized supporters employed and trained by the study team. Assuming approximately equal allocation of participants to study arms, ~7,150 participants will be allocated to the support arms. With a similar uptake of support as the feasibility trials, we would estimate that between 3,575 and 5,000 of these participants will take up the offer of support requiring between one and three 10-min phone calls each. For the feasibility trial eight supporters delivered all of the support and all reported having additional capacity, so although we will of course need to scale up the numbers of supporters, this should not be an unfeasibly large number required. This is especially the case given that participants will be recruited over a period of 2 years compared to 3 months in the feasibility trial, so the need for/provision of this support will be spread over a much longer period. Furthermore, as the support model primarily delivers support within the first 12 weeks of participants' use of Active Brains, some participants will reach the end of their support window by the time newly recruited participants begin theirs, meaning that the same supporter will be able to provide support for multiple practices without their workload becoming overwhelming. We estimate that around 15 supporters will be sufficient to deliver support to start with and we will have capacity to increase this number if required as more participants are recruited.

There were some indications that those in the “lower cognitive score” trial may have used the additional support more than those in the “higher cognitive score” trial – particularly in terms of having multiple telephone appointments. This might indicate greater perceived need for additional support amongst this group. Regular telephone support is advocated for maintaining engagement with complex interventions for those with cognitive impairment (46). Possible indications of differences in usage between the supported and non-supported groups, suggest that the support may have acted differently in the two trials. In the “lower cognitive score” trial, it appeared that a larger proportion of those in the support group accessed brain training compared to in the non-supported group and also appeared to access the games more frequently. In the “higher cognitive score” trial, although it seemed that similar proportions of the supported and non-supported groups accessed brain training, individuals in the supported group appeared to access it more frequently per person. However, in both trials there were early indications that the additional support may have enhanced engagement with brain training. Within the “lower cognitive score” trial, it also appeared that those in the supported group may have been more likely to access the full range of physical activity sub-sections than those in the non-supported group.

### Preliminary indications about intervention outcomes

Potential patterns identified in the primary outcome data suggest that testing of Active Brains in fully powered effectiveness trials is warranted for both groups. In the “lower cognitive score” trial, the indication of higher Baddeley verbal reasoning scores and fewer individuals meeting the AACD/MCI criteria at 1 year was seen in all trial arms including the usual care group. Whilst the apparent improvement in the usual care group may suggest any actual change was not due to the intervention, this might be partly a consequence of the study procedures. All participants entering this trial were advised that their score on the baseline cognitive tests was slightly lower than the average. This may have prompted them to take action over the following year; those allocated to the usual care arm may have sought external advice or interventions beyond the brief advice sheet they were provided with, which may have led to improved scores. This message is no longer presented as it was not deemed acceptable by participants. In the “higher cognitive score” trial, the seemingly minimal change in the Baddeley verbal reasoning scores and proportions meeting the AACD/MCI criteria in the intervention arms compared to indications of sharper decline in the usual care groups gives a provisional indication of a protective effect of Active Brains. It is possible that the higher withdrawal rates from the intervention arms in both trials may account for the patterns seen. However, assuming that the AACD outcomes are missing at random given the observed data, we would expect this apparent protective effect to remain. This would be reduced under the extreme assumption that all of those missing meet the AACD criteria.

### Feasibility and acceptability of study procedures

The findings indicated that the trial procedures were generally feasible, but also highlighted elements that required refining. Whilst there were imbalances of some participant characteristics between trial groups this is not unexpected given relatively small groups and should be overcome in larger main trial samples. Although successful in collecting 94% of the notes review data, our findings indicated that changes were required to facilitate sufficient response to the online Baddeley verbal reasoning task – particularly amongst those in the “lower cognitive score” trial who may have found this more challenging. Cognitive decline may detrimentally affect participant retention or follow-up within research given that it can make completion of research tasks more difficult, time consuming, and frustrating (47). In this case, the effect may be compounded by our primary outcome requiring online completion, therefore not offering the usual paper alternative. However, following recommendations by Mody et al. (47), the subsequent changes to our materials and procedures offer participants more guidance and support about completing the online primary outcomes, and provide further explanation and encouragement. The greater completion of the primary outcome in the usual care groups compared to intervention groups likely reflects a combination of higher withdrawal rates in the intervention arms of both trials comparative to usual care, and possible fatigue with, or overlooking of, study emails within the intervention arms. The changes to automated emails and withdrawal process are anticipated to improve primary outcome completion in the intervention arms.

Recruitment and retention of sufficient numbers of participants within the trials was another important factor for being able to collect sufficient follow-up data. We recruited to target (n=180) in each trial within 3 months from 19 primary care practices in just one Clinical Research Network (CRN) within England. Projecting forward to the main trial, this will inevitably require extensive scaling up of recruitment to reach our target sample sizes of n = 10,940 for the “lower cognitive score” trial and *n* = 10,515 for the “higher cognitive score” trial. These sample sizes have been calculated on the basis of detecting a 5% difference in incidence of dementia at 5 years in the “lower cognitive score” trial, and of detecting a mean difference of 0.1 in the Baddeley Verbal Reasoning score in the “higher cognitive score” trial. They assume 70% completion of primary outcomes at 1 year, and 60% completion at 5 years. Full details of the trial sample size calculations are provided in Supplementary Table 7. Whilst we acknowledge that these are ambitious recruitment targets, we have allowed a 2-year recruitment period, and will be working with all 15 CRNs in England to identify primary care practices to participate across the country. We will also expand recruitment into Wales and Scotland for the main trial. Working with England's CRNs during study set up, we have identified that there are over 2,800 research-active (i.e., already engaged in research study activity) primary care practices in England alone. Given that we estimate the need for ~740 practices (based on having recruited an average of 29 participants per practice in these feasibility trials) this indicates that whilst such large recruitment targets will be challenging, there should be sufficient practices to invite to achieve them.

Across both feasibility trials, withdrawal was relatively low, but disproportionately from the intervention arms. Those in the intervention arms naturally had more contact from the study team and therefore more opportunity to request withdrawal. After randomization, the usual care participants were only contacted when 12-month follow-up was due. We considered whether the higher withdrawal from the intervention arms indicated that Active Brains was too burdensome for participants. However, there was no requirement for participants in the intervention arms to engage any more than they wanted and chose to do so. Whilst they would occasionally receive email reminders about new content or suggestions about features of the intervention to try, there was no obligation for them to act on these, and they also had full control over how many emails they received and could stop these if preferred. The changes aimed to make it clearer and easier for participants to stop engaging with the intervention without leaving the trial, and also reassured them that completion of the primary outcome only was sufficient if that's all they could manage. Whilst we anticipate these changes to improve participant retention and primary outcome completion in the main trials, even in this feasibility study neither trial fell substantially below collection of 70% of the primary outcome follow-up data overall. This was the prior agreed criteria with our funder that may indicate lack of feasibility for proceeding to the main trial unless there was a clear and plausible plan to increase responses rates or reduce missing data.

### Strengths and limitations

The study's parallel design allowed us to explore study objectives amongst older adults with and without existing indications of cognitive decline. This has allowed optimization of the intervention and procedures amongst both groups. We can now trial the intervention to determine its effectiveness for both groups. In-depth qualitative work conducted alongside these feasibility trials will be published separately providing further insight into participants' engagement with Active Brains.

A key limitation of this study was the lack of diversity in our sample with regards to ethnicity and relative deprivation. A predominantly white sample, largely from areas of low relative deprivation may not represent the outcomes or engagement we might have seen with a more diverse sample. It is possible, for example, that a more diverse sample may have different requirements or preferences for intervention support, tailoring, or functionality. Our recruitment region is likely to have contributed to our sample's lack of diversity. The average IMD score of all invitees was 6.9, indicating lower than average relative deprivation amongst all those invited. Furthermore, the South West region of England has the UK's lowest proportion of non-white residents (48). For the main trials, we will employ a nationwide recruitment strategy to encourage invitation of more diverse groups in terms of both ethnicity and relative deprivation. Furthermore, we will consider other strategies to maximize recruitment of a diverse sample– for example, targeting areas with higher proportions of non-white residents and/or higher relative deprivation indices. We will also aim to engage with PPI contributors with a more diverse range of characteristics, perspectives, and experiences to ensure our recruitment strategies are accessible and engaging to a diverse audience. For example, this may include reviewing our recruitment materials and procedures with a more diverse group of PPI contributors to ensure that the content, tone and delivery of these is also appropriate, appealing and relevant for individuals from communities with higher relative deprivation and from a range of ethnic backgrounds. Where possible we will also explore the potential for community-based recruitment routes whereby researchers may be able to visit community groups, networks and institutions to introduce and explain the study, have the opportunity to answer questions and invite people directly.

Inevitably, an intervention that necessitates (even brief or occasional) access to a computer/the internet will not always be accessible to all, and we acknowledge that those from more deprived communities may have disproportionately fewer opportunities access to Active Brains. The process evaluation of the main trials will provide an opportunity to explore the reach of recruitment and sign-up to understand this better in the context of a nationwide recruitment study. However, by trialing such a digital resource for those who can access it, if effective it could potentially free up other “in person” resources for those who cannot. In the meantime, it offers a scalable, relatively low-cost way of identifying which recommendations/strategies etc. may be most beneficial which could then be further developed to be accessible non-digitally too.

## Conclusions

This study investigated whether a multi-domain digital behavior change intervention to protect cognitive health is feasible and acceptable amongst adults aged 60–85 both with and without existing indications of cognitive decline. The proposed trial procedures were largely feasible and confirmed that a nationwide primary care recruitment strategy, whilst challenging, should be a suitable approach. Minor modifications to recruitment and follow-up materials and procedures were deemed important for providing participants with additional support and encouragement to complete the online primary outcome measures. Whilst initial uptake and engagement with the online intervention was modest, it was in line with typical usage of other digital behavior change interventions, and early indications from the descriptive analysis of the primary outcome and behavioral data suggest that further exploration of the potential protective benefits of Active Brains are warranted. Large-scale fully powered effectiveness trials amongst older adults with (*n* = 10,940) and without (*n* = 10,515) indications of existing cognitive decline will now investigate whether Active Brains is effective in reducing cognitive decline.

## Data availability statement

The raw data supporting the conclusions of this article will be made available by the authors, without undue reservation.

## Ethics statement

The studies involving human participants were reviewed and approved by National Health Service Research Ethics Committee (reference 17/SC/0463). The patients/participants provided their written informed consent to participate in this study. Written informed consent was obtained from the individual(s) for the publication of any potentially identifiable images or data included in this article.

## Author contributions

PL and LY conceived of the study and secured funding. PL, LY, RE, and KB led the research and RE drafted the first version of this manuscript with input from SP. RE, MW, EG, SP, KS, FM, JS-B, JD-D, KB, AF, LY, and PL all contributed to the design, iterative development, and conduct of study procedures and collection of study data. JK, VH, and JS managed and coordinated the feasibility trials and provided administrative support. JZ, KS, and JD-D provided technical support and expertise in building and amending the digital intervention. HB and CB provided and coordinated access to the cognitive training and assessment tasks. BS and TB conducted the quantitative analysis. GY and SZ conducted the health economic analysis. TK, SG, NM, SRo, MR, HB, CB, LR, GG, JG, SRa, BG, RP, TS, and JN are members of the research management group and provided regular input on study processes, data, and write-up throughout. All co-authors reviewed, commented on, edited or approved this manuscript.

## Funding

This work was supported by the National Institute for Health and Care Research (NIHR) under its Programme Grants for Applied Research (Reference Number RP-PG-0615-20014).

## Conflict of interest

LY is a member of the NIHR Health Protection Research Unit in Behavioral Science and Evaluation at University of Bristol, and the NIHR ARC West. KB's research portfolio is part funded by NIHR ARC Wessex. The remaining authors declare that the research was conducted in the absence of any commercial or financial relationships that could be construed as a potential conflict of interest.

## Publisher's note

All claims expressed in this article are solely those of the authors and do not necessarily represent those of their affiliated organizations, or those of the publisher, the editors and the reviewers. Any product that may be evaluated in this article, or claim that may be made by its manufacturer, is not guaranteed or endorsed by the publisher.

## Author disclaimer

The views expressed are those of the author(s) and not necessarily those of the NIHR or the Department of Health and Social Care.
