# Supplementary material for: Parallel randomized controlled feasibility trials of the “Active Brains” digital intervention to protect cognitive health in adults aged 60–85
Source: Front Public Health. 2022 Sep 20;10:962873. doi: 10.3389/fpubh.2022.962873 (PMC9530972; doi:10.3389/fpubh.2022.962873)
Supplement: Supplementary file 1 [file Table_1.DOCX]

Supplementary Material

# Supplementary table 1 – supporter feedback and solutions implemented

Whilst most supporters reported the support protocol to be feasible to deliver, several issues and uncertainties were identified that informed amendments to the supporter training materials and the processes though which supporters recorded support provision

| **Problem identified** | **Solution Implemented** |
| --- | --- |
| Support log confusing and complicated to use; did not record all information required | Developed ‘offline’ spreadsheet with input from supporters for recording supporter activity in the main trial |
| Uncertainty about dealing with/responding to specific scenarios/questions:  - how to manage participant expectations about purpose of supporter role  -how to support participants with specific concerns about cognitive training scores  - ensuring information they provided aligned with study materials/documentation | Added corresponding sections to the supporter training materials to provide advice on managing these specific issues  Added participant facing recruitment materials and copies of automated support materials ad appendices to supporter training materials |

# Supplementary table 2 - frequency of brain training-related activity at follow-up in both trials

As well as data about how users engaged with the Brain Training module (Table 2), we also collected data on other brain-training related behaviours. This data was collected only at follow-up to avoid contamination of the usual care group with suggestions about potentially beneficial activities for brain health.

|  | **Lower cognitive score Trial** | | | **Higher cognitive score Trial** | | |
| --- | --- | --- | --- | --- | --- | --- |
| Median (IQR)* | Active Brains  (n=53) | Active Brains + Support  (n=59) | Usual Care (n=68) | Active Brains (n=65) | Active Brains + Support (n=60) | Usual Care (n=55) |
| Crosswords/sudoku/puzzles | 4 (1,5) | 4 (2,5) | 4 (3,5) | 4 (3,5) | 5 (3,5) | 4 (3,5) |
| Read newspapers | 4 (3,5) | 4 (4,5) | 4 (4,5) | 4 (3,5) | 4 (4,5) | 4 (3,5) |
| Read books | 4 (4,5) | 4 (3,5) | 4 (3,5) | 5 (3,5) | 5 (4,5) | 5 (3,5) |
| Board games | 2 (1,3) | 2.5 (1.5,3.5) | 2 (1,3) | 2 (1,3) | 2 (1,3) | 2 (1,3) |
| Visit museum | 2 (1,2) | 2 (2,2) | 2 (1,2) | 2 (2,2) | 2 (1,2) | 2 (1,2) |
| New hobbies | 2 (1,2) | 1 (1,2) | 1 (1,2) | 1 (1,2) | 2 (1,2) | 1 (1,2) |
| Crafts | 1.5 (1,4) | 2 (1,4) | 1 (1,2) | 1 (1,3) | 2 (1,4) | 1 (1,3) |
| Art | 1 (1,2) | 1 (1,2) | 1 (1,2) | 1 (1,2) | 1 (1,2) | 1 (1,1) |
| Social club | 3 (1,3) | 3 (1,3.5) | 1.5 (1,4) | 2 (1,3) | 1 (1,3) | 2 (1,4) |
| Visit friends | 3 (3,4) | 3 (2.5,4) | 4 (3,4) | 3 (3,4) | 4 (2,4) | 3 (3,4) |
| Listen to music | 5 (4,5) | 5 (4.5,5) | 5 (4,5) | 5 (4,5) | 5 (4,5) | 5 (4,5) |
| DIY repairs | 2.5 (2,4) | 3 (2,4) | 3 (2,3) | 3 (2,4) | 3 (2,3) | 3 (2,4) |
| Movies | 3 (2,3) | 3 (2,3) | 3 (3,4) | 3 (2,3) | 3 (3,3) | 3 (2,3) |
| Walking | 4 (3,4) | 4 (3,4) | 4 (2,4) | 4 (3,4) | 4 (3,4) | 4 (3,4) |
| Gardening | 3 (3,4) | 4 (3,4) | 3 (3,4) | 4 (3,4) | 3 (3,4) | 3 (2,4) |
| Group discussion | 2 (1,3) | 2 (1,3) | 2 (1,3) | 3 (1,4) | 2.5 (1,4) | 2 (1,3) |
| Charity work | 2.5 (1,4) | 1.5 (1,4) | 3 (1,4) | 1 (1,3) | 1 (1,4) | 2 (1,4) |
| Play instrument | 1 (1,1) | 1 (1,1) | 1 (1,1) | 1 (1,1) | 1 (1,1) | 1 (1,1) |
| Cooking | 5 (4,5) | 5 (4.5,5) | 5 (4,5) | 5 (3,5) | 5 (4,5) | 5 (4,5) |
| Dancing | 1 (1,2) | 1 (1,1) | 1 (1,2) | 1 (1,2) | 1 (1,1) | 1 (1,2) |
|  |  |  |  |  |  |  |
| Missing activity info (n, %) | 17 (32.1%) | 22 (37.3%) | 12 (17.6%) | 19 (29.2%) | 14 (23.3%) | 5 (9.1%) |

These data show the median frequency with which each group engaged with the relevant activity, with 1 being never, to 5 being every day. Overall, this indicated that reading books was a more common pastime amongst ‘cognitively healthy’ participants, whereas engaging in charity work appeared more common amongst the ‘cognitively impaired’ trial participants. Within the ‘cognitively impaired’ trial, there was some indication that those in the intervention arms more frequently attend social clubs at follow-up.

# Supplementary table 3 - baseline and follow-up brief FFQ (dietary pattern) data in both trials

| **Lower cognitive score Trial** | **Active Brains (n=53)** | | **AB + Support (n=59)** | | **Usual Care (n=68)** | |
| --- | --- | --- | --- | --- | --- | --- |
| **Portions per week (median)** | **Baseline** | **Follow up** | **Baseline** | **Follow up** | **Baseline** | **Follow up** |
| **FFQ – prudent pattern score (mean, SD)** | -0.06(1.19) | 0.25(1.61) | 0.06 (0.87) | -0.04 (0.94) | 0.13 (0.72) | -0.12 (0.65) |
| **Oily fish** | 1 | 1 | 0.5 | 1 | 1 | 1 |
| **Olive oil (one tbsp)** | 3 | 3 | 3 | 3 | 3 | 3 |
| **Almond/hazelnut/walnut** | 0.2 | 0.5 | 0.5 | 0.5 | 1 | 1 |
| Missing FFQ score (n, %) | 1 (1.9%) | 23 (43.4%) | 4 (6.8%) | 24 (40.7%) | 3 (4.4%) | 16 (23.5%) |
| **Higher cognitive score Trial** | **Active Brains (n=65)** | | **AB + Support (n=60)** | | **Usual Care (n=55)** | |
| **Portions per week (median)** | **Baseline** | **Follow up** | **Baseline** | **Follow up** | **Baseline** | **Follow up** |
| **FFQ – prudent pattern score (mean, SD)** | 0.04 (0.86) | 0.08 (1.03) | 0.12 (0.69) | 0.19 (0.85) | 0.00 (0.67) | -0.13 (0.83) |
| **Oily fish** | 1 | 1 | 1 | 1 | 1 | 1 |
| **Olive oil (one tbsp)** | 3 | 3 | 3 | 3 | 3 | 3 |
| **Almond/hazelnut/walnut** | 1 | 1 | 1 | 3 | 0.5 | 3 |
| Missing FFQ score (n, %) | 0 (0.0%) | 21 (32.3%) | 1 (1.7%) | 16 (26.7%) | 0 (0.0%) | 7 (12.7%) |

The FFQ prudent pattern score indicates how close to a ‘healthy diet’ (i.e. one conforming to healthy eating recommendations) an individuals’ food consumption is, with higher scores indicating healthier eating. We also used the FFQ to measure portions per week of specific foods recommended by the intervention as protective of cognitive health.

There was little indication of change in eating behaviours between baseline and follow-up in either trial. However, there appeared to be slight improvement in prudent pattern score (indicating how closely an individual’s food consumption conforms to healthy eating recommendations) in the Active Brains group in the ‘cognitively impaired’ trial.

# Supplementary table 4 - completion of primary outcome broken down by trial and intervention group

|  | **Total**  **n** | **Completed Primary Outcome Measure** | ***%*** |
| --- | --- | --- | --- |
| **Trial:** | | | |
| **Lower cognitive score Trial** | 180 | 121 | *67.2%* |
| **Higher cognitive score Trial** | 180 | 137 | *76.1%* |
| **Intervention group (across both trials):** | | | |
| **Active Brains** | 118 | 80 | *67.8%* |
| **Active Brains plus support** | 119 | 78 | *65.5%* |
| **Usual care** | 123 | 100 | *81.3%* |
| **Total** | 360 | 258 | ***71.7%*** |

# Supplementary table 5 – additional detail of health economics analysis

A large proportion of service usage occurred within primary care (239; 66%) with only a few cases in NHS phone calls and walk-in centres. Three quarters of patients took at least one medication. Outpatient appointments were fairly common and relevant A&E visits and hospital admission less common.

| **Type of service use** | **Number of services (%)** | **Mean (STD)** |
| --- | --- | --- |
| Primary care consultation | 239 (66%) | 3.26 (2.15) |
| GP home visit | 3 (1%) | 1 (0) |
| NHS phone call | 10 (3%) | 1.3 (0.67) |
| Medication | 265 (74%) | 3.98 (2.95) |
| Outpatient | 96 (27%) | 1.8 (1.19) |
| A&E | 22 (6%) | - 1. 0.21) |

# Supplementary table 6 - Problems and solutions identified for the screening process

| **Screening step** | **Problem Identified** | **Solution implemented** |
| --- | --- | --- |
| Screening by practice staff following database search to exclude ineligible participants on the basis of:   - recorded diagnosis of dementia; - diagnosis of a serious mental illness (e.g. schizophrenia, major uncontrolled depression); - being terminally ill/ in receipt of palliative care; - someone else within their household already being invited to participate in the study; - any other reason the GP felt a specific participant was ineligible | One practice did not operationalise screening criteria to prevent multiple people per household being invited to participate (to prevent risk of contamination of the control groups should different household members be randomised to different trial arms)  One practice reported concerns about operationalising ‘one participant per household’ criteria in terms of having to field calls from patients who didn’t understand why they, but not their spouse, had been invited (or vice versa). | We modified the instruction documentation for practices to ensure this was clearer and members of the trial management team reiterated this point in email or verbal communication with practices during their setup.  We modified our recruitment materials to make it clearer that only one person per household is able to participate and that, where both may be eligible, the invitee was chosen at random. |
| Participant-completed online screening questions on the Active Brains website to screen out ineligible invitees:   - Godin Leisure Time Exercise Questionnaire (GLTEC (1)) to assess baseline physical activity. Participants scoring >30 were identified as ‘highly physically active’ and excluded in line with previously applied thresholds (2); - confirmation of age; - dementia diagnosis status; - participation in any other intervention studies. | Based on our International Physical Activity Questionnaire (brief version for older adults – IPAQ- E; (3)) and qualitative process data, our samples appeared to include individuals who reported high levels of physically activity at baseline when these individuals should have been screened out.  Could imply a failure of the GLTEQ to identify highly active individuals (despite successfully screening out 267), or might demonstrate participant over-reporting of physical activity on the IPAQ-E measure, or possibly a combination of the two. | We discussed replacing either or both of these self-report tools, but opted not to for several reasons:   1. Without sufficient funds for objective monitoring of physical activity in such a large sample, we were reliant on self-report measures. 2. GLTEQ intentionally chosen as a short and simple measure that could be used for screening, whereas IPAQ-E deemed to have greater granularity to allow identification of small changes in physical activity – both factors remained important. 3. Over-estimation/ over-reporting of physical activity behaviour is well recognised (4). As such, we considered it better to continue to be potentially over-inclusive of higher physical activity levels when these may often be over-estimated, rather than exclude those who may potentially benefit. |
| Baddeley verbal reasoning task to determine eligibility for either the ‘lower cognitive score’ or ‘higher cognitive score’ trial | Online message presented to individuals meeting the criteria for ‘lower cognitive score’ trial designed to inform them that whilst their score on this occasion was slightly below the average that this should not, on its own, be cause for alarm and that there may be many other reasons for this.  Despite this message being carefully developed and piloted, with PPI contributors and participants, feedback from qualitative process interviews and email correspondence from participants indicated that this message was frequently experienced as demotivating, patronising and even slightly concerning. | We took the decision to remove this message; those eligible for the ‘lower cognitive score’ trial are no longer informed about their relative lower score on the initial cognitive assessment. However, messages remain throughout the intervention content encouraging individuals to report any ongoing concerns about their cognitive performance to their GP. |

1. Shephard R. Godin leisure-time exercise questionnaire. Med Sci Sports Exerc. 1997;29(6):S36-S8.
2. Cooper, A. J. M., Dearnley, K., Williams, K. M., Sharp, S. J., van Sluijs, E. M. F., Brage, S., Sutton, S., & Griffin, S. J. Protocol for Get Moving: a randomised controlled trial to assess the effectiveness of three minimal contact interventions to promote fitness and physical activity in working adults. BMC public health. 2015; 15(1), 296. <https://doi.org/10.1186/s12889-015-1654-0>
3. Hurtig-Wennlof A, Hagstromer M, Olsson LA. The International Physical Activity Questionnaire modified for the elderly: aspects of validity and feasibility. Public health nutrition. 2010;13(11):1847-54.
4. Falck, R. S., McDonald, S. M., Beets, M. W., Brazendale, K., & Liu-Ambrose, T. Measurement of physical activity in older adult interventions: a systematic review. Br J Sports Med. 2016; 50(8), 464-470. <https://doi.org/10.1136/bjsports-2014-094413>

**Supplementary table 7 – Main Trial Power Calculations**

| 1. **Lower Cognitive Score trial** | | | **Number of MCI/AACD participants per trial arm required** | | **Total number of MCI /AACD participants required** | **Total number of participants per trial arm** | | **Total** |
| --- | --- | --- | --- | --- | --- | --- | --- | --- |
| **Primary outcome measures** | | **Cognitive Decline – Baddeley Verbal reasoning** (alpha = 0.025, 80% power to detect standardised effect of 0.15) | 847 of each | | 2541 of each | 1694 | | 5082 |
|  |  | **Progression to Dementia at 5 years**  (Assuming 5% progression rate – alpha = 0.05, 80% power to detect 5% diff after 5 years) | 1094 of each | | 3282 of each | 2188 | | 6564 |
| **For 1 Year Follow-up** | | | | | | | | |
| **Baddeley verbal reasoning - assuming 70% follow-up** | | | 1210 of each | | 3630 of each | 2420 | | 7260 |
| **For 5 Year Follow-up** | | | | | | | | |
| **Baddeley Verbal reasoning – assuming 60% follow up** | | | 1412 of each | | 4235 of each | 2824 | | 8470 |
| **Progression to dementia – assuming 60% follow-up** | | | 1823  of each | | 5470 of each | 3647 | | 10940 |
| 1. **Higher Cognitive Score trial** | | | | **Total number of participants per trial arm** | | | **Total** | |
| **Primary outcome measure** | **Cognitive Decline – Baddeley Verbal reasoning** (alpha = 0.05, 90% power to detect standardised mean difference of 0.1 in Baddeley) | | | 2103 | | | 6309 | |
| **For 1 Year Follow-up** | | | | | | | | |
| **Baddeley verbal reasoning - assuming 70% follow-up** | | | | 3005 | | | 9013 | |
| **For 5 Year Follow-up** | | | | | | | | |
| **Baddeley Verbal reasoning – assuming 60% follow up** | | | | 3505 | | | 10515 | |

**Explanatory notes:**

1. ***Lower Cognitive Score trial***
2. **Baddeley reasoning test (Primary outcome)**

For the primary outcome of cognitive decline we assume an alpha of 0.025 and 80% power to detect a standardised effect of 0.15. This requires 847 per group for the MCI subgroup and the same again for the AACD subgroup – 2541 of each in total.

1. For primary outcome at one-year follow-up:

Assuming that 70% will be available to provide follow-up at 1 year, we will need 3630 participants with MCI and the same with AACD, or 7260 in total.

1. For primary outcome at five-year follow-up:

Assuming that 60% will be available to provide follow-up within 5 years, this rises to 4235 participants with MCI and the same with AACD per group or 8470 in total.

1. **Progression to dementia**

Although the reduction in cognitive decline as assessed using the Baddeley reasoning test is the primary outcome at one-year, the limiting factor in the sample size calculation for those with cognitive impairment is the progression to dementia at five-years. We assume a 5% annual progression rate to dementia among those who are cognitively impaired. With alpha 0.05 and 80% power, we aim to detect a difference of 5% after 5 years (20% intervention and 25% control) in those diagnosed with dementia. This means we would need 1094 in each of the MCI and AACD groups or a total of 2188. Assuming that 60% will be available to provide follow up in 5 years requires 3647 in each of the 3 study groups or 10940 in total.

1. ***Higher Cognitive Score trial***

**Baddeley reasoning test (Primary outcome)**

We assume few of those who have no cognitive impairment at baseline will develop dementia, and that we might find a smaller effect of the intervention. For the primary outcome of cognitive decline we assume an alpha of 0.05 and 90% power to detect a standardised mean difference of 0.1 in the Baddeley. This requires 2103 per group.

1. For primary outcome at one-year follow-up:

Assuming that 70% will be available to provide follow-up at 1 year, we will need 3004 per group, or 9013 in total.

1. For primary outcome at five-year follow-up:

Assuming that 60% will be available to provide follow-up within 5 years, that rises to 3505 per group or 10,515 in total.

Therefore, in order to be powered to detect an effect at 5-year follow-up:

- In patients with cognitive decline we will recruit a minimum of 3647 patients to each of the three study groups (10,940 total); there will be a mixture of patients with MCI and AACD.
- In patients without cognitive decline we will recruit a minimum of 3505 patients to each of the three study groups (10,515 total).

This means our total sample size across both trials will be a minimum of 21,455 participants in the trials.
